# Symptom Clusters and Symptom Burden in Cancer Survivors

**DOI:** 10.1002/cam4.71653

**Published:** 2026-03-05

**Authors:** Marilyn J. Hammer, Carolyn Harris, Rachel Pozzar, Karin Snowberg, Steven M. Paul, Bruce A. Cooper, Maura E. Abbott, Susan Chang, Stacey Kenfield, Erin Van Blarigan, Katherine Van Loon, Jon D. Levine, Christine Miaskowski

**Affiliations:** ^1^ Dana‐Farber Cancer Institute and Northeastern University Boston Massachusetts USA; ^2^ School of Nursing, University of California San Francisco California USA; ^3^ School of Medicine, University of California San Francisco California USA; ^4^ Columbia University Medical Center New York New York USA

**Keywords:** cancer, exploratory factor analysis, patient reported outcomes, survivors, symptom clusters, symptoms

## Abstract

**Context:**

Limited information is available on the symptom burden and symptom clusters in cancer survivors.

**Objectives:**

Describe the occurrence, severity, and distress of 44 symptoms; determine risk factors associated with a higher symptom burden; and evaluate for symptom clusters using symptom occurrence rates.

**Methods:**

Survivors (*n* = 1147) were recruited using an online survey. Symptom burden and symptom clusters were assessed using the Memorial Symptom Assessment Scale that included 44 symptoms and evaluated occurrence, severity, and distress. Simultaneous multivariable linear regression analysis was performed to determine risk factors associated with a higher symptom burden. Exploratory factor analysis was used to identify symptom clusters using ratings of symptom occurrence.

**Results:**

Survivors reported an average ten concurrent symptoms. Survivors who are younger, female, with a higher comorbidity burden, evidence of metastatic disease, and a poorer functional status were at increased risk for a higher symptom burden. Six symptom clusters were identified (i.e., psychological cluster, cancer and treatment‐related cluster, respiratory cluster, pain cluster, weight loss cluster, epithelial cluster).

**Conclusion:**

Additional research is warranted to confirm the prevalence rates for the various symptoms and symptom clusters; identify additional risk factors for a higher symptom burden; and determine the underlying mechanisms for the symptom clusters. Future studies need to develop and test targeted interventions for each of the symptom clusters within and across various types of cancer.

## 1 Introduction

In the United States, the estimated number of cancer survivors is 18.1 million, which represents 5.4% of the population. With advances in treatment, this number is projected to increase to 21.6 million by 2030 and to 26 million by 2040 [1]. While prolongation of life is a major achievement, many cancer survivors experience long‐term effects from the disease and its treatments. In reports from the Office of Cancer Survivorship at the National Cancer Institute [2, 3, 4], emphasis was placed on the need to evaluate the symptom burden of cancer survivors, particularly in the context of newer treatments.

Reviews of symptom burden in cancer survivors suggest that the most common symptoms reported are pain, fatigue, and depression and that symptoms persist for more than 10 years [5, 6]. Recent work focused on an evaluation of symptom clusters in cancer survivors [7, 8, 9, 10, 11]. The rationale for an evaluation of symptom clusters is that survivors rarely experience a single symptom, and previous research demonstrated that relationships exist between/among various symptoms [12].

To date, the evidence on symptom clusters in cancer survivors is somewhat disparate [7, 8, 9, 10, 11]. While two studies assessed survivors with various types of cancer [9, 11], three included only survivors with breast cancer [7, 8, 10]. Equally important, the number of symptoms included in these analyses was highly variable. Therefore, definitive conclusions cannot be made about the most common symptom clusters in cancer survivors.

Given the growing number of cancer survivors and the lack of a comprehensive evaluation of their symptom burden, the purposes of this study, in a sample of cancer survivors (*n* = 1147), were to describe the occurrence, severity, and distress of 44 symptoms; determine risk factors associated with a higher symptom burden; and evaluate for symptom clusters using symptom occurrence rates.

## 2 Methods

### 2.1 Sample and Settings

Survivors were recruited from a registry of individuals who participated in our previous symptom management studies; from electronic health record searches for patients with cancer diagnoses at University of California, San Francisco (UCSF) and Mount Sinai Medical Center and Columbia University Medical Center in New York City; and from the Dr. Susan Love Foundation for Breast Cancer Research. While several definitions of cancer survivors exist, this study used the American Cancer Society's definition (i.e., “anyone who has ever been diagnosed with cancer no matter where they are in the course of their disease”) [13].

Survivors received an email with a brief explanation of the study and a link that directed them to the study's enrollment page. This page explained the study's purpose, time frame for survey completion, and information about participating in research. This study was exempt from requiring written informed consent by the UCSF Institutional Review Board and from the other sites. Survivors were included if they were ≥ 18 years of age; were able to read, write, and understand English; had a diagnosis of cancer; were able to complete study questionnaires online; and by completing the survey consented to participate. Of the 1908 survivors who began the survey, 1147 completed the information that is presented in this paper.

### 2.2 Recruitment and Survey Administration

Emails were sent to potential survivors from May 27, 2020 to February 22, 2021. Survivors were asked to complete the survey within two weeks. After 14 days, one reminder was sent to the survivors who did not respond to the initial email request.

Survivors were asked to answer all of the survey questions in relationship to their experiences in the past 14 days. The entire survey took approximately 60 min to complete. Survivors were advised that doing the survey in one sitting was preferable but to take as many breaks as needed. All of the instruments were completed online using REDCap [14].

### 2.3 Instruments

#### 2.3.1 Demographic and Clinical Characteristics

Survivors completed a demographic questionnaire, Karnofsky Performance Status (KPS) scale [15], Self‐Administered Comorbidity Questionnaire (SCQ) [16], and International Physical Activity Questionnaire [17]. In addition, survivors responded to questions about their height and weight, cancer diagnosis, previous and current cancer treatments, and presence of metastatic disease.

#### 2.3.2 Symptom Measure

A modified version of the 32‐item Memorial Symptom Assessment Scale (MSAS) was used to evaluate the occurrence, severity, and distress of 44 common symptoms associated with cancer and its treatment [18]. “Changes in skin” was deleted from the original list of 32 symptoms and the following symptoms were added: joint pain; muscle aches and pain; dry skin; ringing in your ears; weight gain; calluses on hands and/or feet; burning or redness of the eyes; changes in nails (brittle); abnormal sensations or urge to move your legs; skin rash; changes in your sense of smell; swollen, red, and painful sores around the fingernails and/or toenails; and long thick eyelashes. These symptoms were added to include common symptoms that were not on the original MSAS when it was developed in 1994. Survivors reported whether they had experienced each symptom in the past 14 days (i.e., symptom occurrence). If they had experienced the symptom, they were asked to rate its severity and distress. Severity was rated using a four‐point Likert scale (i.e., 1 = slight, 2 = moderate, 3 = severe, 4 = very severe). Distress was rated using a five‐ point Likert scale (i.e., 0 = not at all, 1 = a little bit, 2 = somewhat, 3—quite a bit, 4 = very much).

### 2.4 Data Analyses

Descriptive statistics were calculated on sample characteristics, as well as symptom scores using IBM SPSS Statistics version 29 (IBM Corporation, Armonk, NY). Demographic and clinical characteristics, that were significantly associated with the number of MSAS symptoms in the bivariable analyses, were evaluated in a simultaneous multivariable linear regression analysis to determine which characteristics were associated with a higher number of MSAS symptoms (i.e., a higher symptom burden). For all the tests, a *p*‐value of < 0.05 was considered statistically significant.

To identify the symptom clusters based on occurrence rates, exploratory factor analysis (EFA) was done using Mplus [19]. Symptoms with an occurrence rate of ≥ 10% were included in the EFA (i.e., 37 out of 44). Factor loadings were considered meaningful if the loading was ≥ 0.40 [19]. Factors were adequately defined if at least two symptoms had loadings of ≥ 0.40 [20]. Items were allowed to cross‐load if they fell within our preset criteria of ≥ 0.40. Tetrachoric correlations were used to create the matrix of associations for the occurrence items [19]. Simple structures for the EFAs were estimated using the method of unweighted least squares with geomin (i.e., oblique) rotation [19].

Factor solutions were estimated for two through eight factors. Factor solution with the greatest interpretability and clinical meaningfulness was selected, given that it met the criteria set for evaluating simple structure. Clusters were named based on the symptoms with the highest factor loadings and the majority of the symptoms in the cluster.

## 3 Results

### 3.1 Demographic and Clinical Characteristics

Of the 1147 survivors who completed the study questionnaires, 83.9% were female, 84.9% self‐identified as White, 67.5% were married or partnered, and 43.7% were employed. Survivors were 62.3 (±11.3) years of age, had a SCQ score of 4.1 (±3.6), a KPS score of 90.9 (±10.6), and a median time since diagnosis of 6.2 years (mean = 8.6 (±7.9) years; range 0.4–55.6 years). The most common comorbidities were high blood pressure (32.8%), back pain (32.8%), osteoarthritis (29.0%), and depression (26.7%). The majority of the patients had breast cancer (68.7%), 27.3% had metastatic disease, and 43.9% were receiving active treatment (Table 1).

**TABLE 1:** Characteristics of the cancer survivors (*n* = 1147).

Characteristic	Mean (SD)
Age (years)	62.3 (11.3)
Number of people in your household including yourself	2.1 (1.1)
Body mass index (kg/m^2^)	27.0 (6.0)
Karnofsky Performance Status score	90.9 (10.6)
Number of comorbidities	2.0 (1.6)
Self‐administered Comorbidity Questionnaire score	4.1 (3.6)
Time since cancer diagnosis (years)	8.6 (7.9)
Time since diagnosis (median, years)	6.2
Number of previous cancer treatments	2.7 (1.2)
Number of current cancer treatments	0.5 (0.7)
Total metabolic equivalents^a^	3060.0 (3200.2)
Number of MSAS symptoms (out of 44)	10.0 (5.8)
	**% (*n*)**
Female (% yes)	83.9 (945)
Lives alone (% yes)	24.1 (276)
Married/partnered (% yes)	67.5 (772)
Race	
White	84.9 (971)
Non‐White	15.1 (173)
Highest level of education	
Grade school	0.4 (5)
High school	3.1 (35)
Some college	16.1 (183)
College graduate	24.5 (279)
Some graduate school	9.7 (111)
Advanced degree	46.2 (527)
Currently employed (% yes)	43.7 (498)
Annual household income	
< $20,000	3.8 (35)
$20,000–$59,000	18.5 (172)
$60,000–$100,000	23.1 (215)
> $100,000	54.7 (509)
Level of exercise	
Low	24.6 (282)
Moderate	37.5 (430)
High	37.9 (435)
Chronic conditions (% yes)	
Heart disease	11.3 (127)
High blood pressure	32.8 (373)
Lung disease	5.8 (65)
Diabetes	6.5 (73)
Ulcer or stomach disease	4.4 (49)
Kidney disease	2.9 (33)
Liver disease	2.8 (31)
Anemia or blood disease	8.5 (95)
Depression	26.7 (299)
Osteoarthritis	29.0 (327)
Back pain	32.8 (369)
Rheumatoid arthritis	3.4 (37)
Cancer diagnosis (% yes)	
Breast	68.7 (773)
Gastrointestinal	3.9 (44)
Head and neck	1.1 (12)
Multiple myeloma	2.0 (23)
Leukemia	1.4 (16)
Lung	0.6 (7)
Malignant melanoma	0.4 (5)
Lymphoma	1.4 (16)
Gynecological	2.0 (22)
Prostate	6.4 (72)
Brain	0.4 (4)
Multiple or other	11.6 (131)
Presence of metastatic disease (% yes)	27.3 (298)
Currently receiving cancer treatment (% yes)	43.9 (504)

Abbreviations: kg, kilograms; m^2^, meters squared; SD, standard deviation.

^a^ Assessed using the International Physical Activity Scale.

### 3.2 Symptom Occurrence, Severity, and Distress

The mean number of MSAS symptoms was 10.0 (±5.8). Table 2 lists the occurrence, severity, and distress scores for the 44 MSAS symptoms. As shown in Table 3, the four symptoms that occurred in over 50% of the survivors were pain, difficulty sleeping, lack of energy, and worrying. The four symptoms with the highest severity ratings were: problems with sexual interest or activity, changes in your sense of smell, I don't look like myself, and difficulty sleeping. The four symptoms with the highest distress ratings were: hair loss, weight gain, problems with sexual interest or activity, and I don't look like myself.

**TABLE 2:** Symptom occurrence, severity, and distress ratings in cancer survivors (*n* = 1147).

Symptom^a^	Occurrence^b^		Severity with zeros		Severity without zeros^c^		Distress^d^	
	%	*n*	Mean	SD	Mean	SD	Mean	SD
Pain	63.7	728	1.21	1.06	1.90	0.67	1.90	0.93
Difficulty sleeping	58.4	667	1.22	1.21	2.10	0.82	2.02	1.06
Lack of energy	57.4	655	1.14	1.14	1.99	0.76	2.00	1.07
Worrying	50.7	574	0.95	1.09	1.88	0.79	1.77	0.96
Joint pain	47.7	542	0.92	1.09	1.94	0.73	1.83	0.97
Muscle aches and pain	46.3	526	0.83	1.02	1.80	0.71	1.59	1.01
Difficulty concentrating	45.6	520	0.82	1.02	1.79	0.73	1.87	1.01
Feeling drowsy	45.4	514	0.78	0.98	1.72	0.70	1.23	1.05
Feeling sad	44.6	507	0.81	1.04	1.82	0.76	1.74	1.01
Feeling irritable	37.7	426	0.64	0.94	1.71	0.73	1.67	0.97
Numbness/tingling in hands/ft	37.5	426	0.67	0.99	1.79	0.77	1.61	1.05
Feeling nervous	34.2	390	0.62	0.97	1.80	0.78	1.81	0.97
Dry skin	33.1	376	0.56	0.90	1.70	0.71	1.19	0.94
Problems with sexual interest or activity	31.6	355	0.88	1.42	2.80	1.01	2.22	1.22
Sweats	25.3	289	0.50	0.94	1.96	0.78	1.73	1.11
Ringing in your ears	23.4	266	0.42	0.85	1.82	0.74	1.37	0.94
Itching	21.1	240	0.38	0.82	1.79	0.81	1.62	1.06
Feeling bloated	20.2	229	0.38	0.84	1.89	0.79	1.83	1.11
Constipation	20.1	223	0.36	0.81	1.82	0.77	1.62	1.09
Weight gain	18.3	208	0.26	0.63	1.44	0.66	2.23	1.27
Dry mouth	17.9	204	0.34	0.80	1.87	0.86	1.35	1.03
I don't look like myself	17.8	202	0.38	0.91	2.14	0.94	2.22	1.15
Calluses on hands and/or feet	15.7	177	0.26	0.66	1.64	0.73	0.94	0.99
Burning and redness of the eyes	15.2	173	0.25	0.65	1.62	0.72	1.54	0.96
Dizziness	14.9	169	0.23	0.62	1.54	0.76	1.52	0.99
Cough	13.4	153	0.18	0.52	1.38	0.60	1.08	1.04
Changes in nails (brittle)	13.4	151	0.24	0.66	1.77	0.74	1.42	0.96
Abnormal sensations or urge to move your legs	12.8	146	0.24	0.69	1.86	0.83	1.68	1.08
Swelling of arms or legs	12.3	140	0.21	0.62	1.72	0.78	1.85	1.03
Lack of appetite	12.0	136	0.21	0.61	1.74	0.68	1.10	0.97
Hair loss	12.0	137	0.22	0.70	1.85	1.00	2.43	1.24
Diarrhea	11.5	130	0.21	0.65	1.86	0.78	1.72	1.11
Weight loss	10.8	123	0.12	0.38	1.15	0.36	0.30	0.70
Shortness of breath	10.8	123	0.17	0.52	1.54	0.63	1.59	0.90
Skin rash	10.8	122	0.17	0.56	1.61	0.76	1.57	1.00
Nausea	10.4	118	0.17	0.55	1.62	0.74	1.67	1.12
Problems with urination	9.8	112	0.19	0.62	1.90	0.83	1.91	1.06
Mouth sores	6.4	73	0.10	0.45	1.64	0.79	1.55	1.07
Change in the way food tastes	4.7	53	0.10	0.47	2.08	0.83	1.87	1.04
Difficulty swallowing	3.7	42	0.06	0.37	1.74	0.94	1.55	1.02
Changes in your sense of smell	2.0	23	0.05	0.35	2.26	1.10	1.61	1.27
Swollen red and painful sores around the fingernails and/or toenails	2.0	23	0.03	0.24	1.52	0.73	1.65	0.89
Vomiting	1.8	20	0.03	0.23	1.60	0.75	2.10	1.02
Long and thick eyelashes	1.5	17	0.02	0.21	1.56	0.89	0.18	0.39

*Note:* Symptom severity and distress scores are for those patients who reported the occurrence of the symptom.

Abbreviation: SD, standard deviation.

^a^ Symptoms are from the Memorial Symptom Assessment Scale with deletion of changes in skin and the addition of the following symptoms: joint pain, muscle aches and pain, dry skin, ringing in your ears, weight gain, calluses on hands and/or feet, burning or redness of the eyes, changes in nails (brittle), abnormal sensations or urge to move your legs, skin rash, changes in your sense of smell, swollen, red, and painful sores around the fingernails and/or toenails, and long thick eyelashes.

^b^ Symptoms are listed in descending order of occurrence.

^c^ Severity ratings without zeros: 1 = slight, 2 = moderate, 3 = severe, 4 = very severe.

^d^ Distress ratings: 0 = not at all, 1 = a little bit, 2 = somewhat, 3 = quite a bit, 4 = very much.

**TABLE 3:** Rankings of symptom occurrence, severity, and distress.

Rank	Occurrence^b^		Severity^c^		Distress^d^	
	Symptom^a^	%	Symptom^a^	Mean (SD)	Symptom^a^	Mean (SD)
1	Pain	63.7	Problems with sexual interest or activity	2.80 (1.01)	Hair loss	2.43 (1.24)
2	Difficulty sleeping	58.4	Changes in your sense of smell	2.26 (1.10)	Weight gain	2.23 (1.27)
3	Lack of energy	57.4	I don't look like myself	2.14 (0.94)	Problems with sexual interest or activity	2.22 (1.22)
4	Worrying	50.7	Difficulty sleeping	2.10 (0.82)	I don't look like myself	2.22 (1.15)
5	Joint pain	47.7	Chang in way food tastes	2.08 (0.83)	Vomiting	2.10 (1.02)
6	Muscle aches and pain	46.3	Lack of energy	1.99 (0.76)	Difficulty sleeping	2.02 (1.06)
7	Difficulty concentrating	45.6	Sweats	1.96 (0.78)	Lack of energy	2.00 (1.07)
8	Feeling drowsy	45.4	Joint pain	1.94 (0.73)	Problems with urination	1.91 (1.06)
9	Feeling sad	44.6	Pain	1.90 (0.67)	Pain	1.90 (0.93)
10	Feeling irritable	37.7	Problems with urination	1.90 (0.83)	Difficulty concentrating	1.87 (1.01)
10	—	—	—	—	Change in way food tastes	1.87 (1.04)

*Note:* Symptom severity and distress scores are for those patients who reported the occurrence of the symptom.

Abbreviation: SD, standard deviation.

^a^ Symptoms are from the Memorial Symptom Assessment Scale with deletion of changes in skin and the addition of the following symptoms: joint pain, muscle aches and pain, dry skin, ringing in your ears, weight gain, calluses on hands and/or feet, burning or redness of the eyes, changes in nails (brittle), abnormal sensations or urge to move your legs, skin rash, changes in your sense of smell, swollen, red, and painful sores around the fingernails and/or toenails, and long thick eyelashes.

^b^ Symptoms are listed in descending order of occurrence.

^c^ Severity ratings without zeros: 1 = slight, 2 = moderate, 3 = severe, 4 = very severe.

^d^ Distress ratings: 0 = not at all, 1 = a little bit, 2 = somewhat, 3 = quite a bit, 4 = very much.

### 3.3 Risk Factors for Higher Symptom Burden

Eleven demographic and clinical characteristics, that were significantly associated with the total number of MSAS symptoms, were entered into the multivariable linear regression analysis (Table 4). Six characteristics (i.e., younger age, being female, having a higher comorbidity burden, having a poorer functional status, had received a higher number of cancer treatments, and evidence of metastatic disease) were associated with self‐reports of a higher number of MSAS symptoms. Final model explained 33.1% of the total variance.

**TABLE 4:** Results of the simultaneous multivariable linear regression analysis of demographic and clinical characteristics associated with a higher symptom burden (*n* = 984).

Total number of symptoms					
Characteristic	*R* ^2^	*r*	*β*	*R* ^2^‐change (sr^2^)	*p*
Overall	0.331	—	—	—	< 0.001
Age	—	−0.153	−0.100	0.008	< 0.001
Female	—	0.163	0.185	0.029	< 0.001
Body mass index	—	0.139	−0.007	0.00005	0.797
Total metabolic equivalents^a^	—	−0.106	0.010	0.0001	0.705
Comorbidity burden^b^	—	0.401	0.264	0.048	< 0.001
Functional status^c^	—	−0.478	−0.326	0.074	< 0.001
Time since cancer diagnosis (years)	—	−0.088	−0.051	0.002	0.090
Number of previous cancer treatments	—	0.130	0.057	0.003	0.047
Number of current cancer treatments	—	0.171	0.026	0.0001	0.636
Evidence of metastatic disease	—	0.189	0.067	0.004	0.022
Currently receiving cancer treatment	—	0.162	−0.021	0.0001	0.693

^a^ Assessed using the International Physical Activity Scale.

^b^ Self‐Administered Comorbidity Questionnaire score.

^c^ Karnofsky Performance Status Scale score.

### 3.4 Symptom Clusters

Based on the EFA, a six‐factor solution was selected using the symptom occurrence data (Table 5). The symptom clusters were named based on the core symptoms within each cluster (i.e., psychological cluster, cancer and treatment‐related cluster, respiratory cluster, pain cluster, weight loss cluster, epithelial cluster).

**TABLE 5:** Symptom clusters in cancer survivors using occurrence rates^a^.

Cluster	Symptoms	Factor loading
Psychological cluster	Feeling sad	0.857
	Worrying	0.853
	Feeling nervous	0.837
	Feeling irritable	0.784
	Difficulty concentrating	0.586
Cancer and treatment‐related cluster	I don't look like myself	0.808
	Hair loss	0.594
	Changes in nails (brittle)	0.507
	Sweats	0.497
	Weight gain	0.481
	Feeling bloated	0.455
	Nausea	0.437
	Problems with sexual interest or activity	0.402
Respiratory cluster	Shortness of breath	0.630
	Cough	0.626
Pain cluster	Joint pain	0.836
	Pain	0.797
	Muscle aches and pain	0.677
Weight loss cluster	Weight loss	0.718
	Lack of appetite	0.705
	Weight gain	−0.642
Epithelial cluster	Skin rash	0.700
	Itching	0.681
	Dry skin	0.523
	Calluses on hands and/or feet	0.424

^a^ Extraction method: unweighted least squares. Rotation method: Geomin (oblique) rotation.

## 4 Discussion

This study is the first to perform a comprehensive evaluation of multiple dimensions of the symptom experience in cancer survivors. Given that the original MSAS was developed in 1994 [18] and revised by our research team in 2014 [21], symptoms needed to be added that evaluated the effects of hormonal therapies (e.g., joint pain) and targeted and immunotherapies (i.e., a variety of dermatologic toxicities). Given the relatively large sample size and the comprehensive evaluation of the multiple dimensions of the symptom experience, these results can serve as “benchmark data” for future studies.

The mean number of 10 symptoms is slightly lower than the mean of 14 symptoms reported by patients undergoing chemotherapy [22]. However, symptom burden remains high in a cohort of survivors who were over eight years from their cancer diagnosis. Consistent with previous reports [23, 24], the four symptoms with the highest occurrence rates and that occurred in over 50% of the survivors were pain, difficulty sleeping, lack of energy, and worrying. While the impact of pain, fatigue, and sleep disturbance were studied extensively as part of a prespecified psychoneurological symptom cluster [24, 25, 26, 27, 28, 29, 30, 31], depression was the “psychological symptom” that was most often included in this cluster. While in the current study, feeling sad (i.e., a proxy for depressive symptoms) was assessed using the MSAS (i.e., 44.6% of survivors reported this symptom), the occurrence of worrying was higher in the current sample (50.7%). This finding may be related to the fact that these data were collected during the COVID‐19 pandemic. However, in four studies of symptom clusters in cancer survivors [8, 9, 10, 11], anxiety was a highly prevalent symptom. Additional research is warranted on the causes and consequences of both anxiety and depression in cancer survivors because 26.7% of the survivors in this study self‐reported a diagnosis of depression.

Consistent with previous findings [22, 32], the symptoms with the highest occurrence rates were not the most severe and/or distressing (Table 3). Of note, pain, difficulty sleeping, and lack of energy were the only symptoms that were in the top ten across the three dimensions of the symptom experience. Given their high prevalence rates and moderate levels of severity and distress, clinicians need to perform routine assessments of these symptoms and initiate pharmacologic and nonpharmacologic interventions.

### 4.1 Risk Factors for a Higher Symptom Burden

Of the 11 characteristics included in the regression analysis, six risk factors were significantly associated with a higher symptom burden (i.e., younger age, being female, having a higher comorbidity burden, having a poorer functional status, having received a higher number of cancer treatments, and evidence of metastatic disease). In terms of demographic characteristics, the associations between younger age [6, 33] and being female [34, 35] are consistent with previous studies. In terms of a higher comorbidity burden, while this association is consistent with previous reports [6, 33], it should be noted that 57.1% of the survivors in the current study met the criteria for multimorbidity (i.e., two or more coexisting conditions in the same individual) [36]. In addition, the most common comorbid conditions reported by the survivors in the current study (i.e., high blood pressure, depression, back pain, osteoarthritis) are consistent with findings from a scoping review of multimorbidity in people living with and beyond cancer [37]. Given that pain, fatigue, sleep disturbance, depression, and anxiety are associated with both cancer and these common comorbid conditions, oncology clinicians and primary care providers need to collaborate to optimize the management of these conditions and associated symptoms.

In terms of cancer and associated treatment characteristics, while currently receiving cancer treatment was not significant in the multivariable model, receipt of a higher number of previous treatments was associated with a higher symptom burden. While details of specific types of treatments were not obtained in this study, the number of previous treatments ranged from 0 to 6 (mean = 2.7 [±1.2]). While no studies were identified that reported on this association, these results suggest that multiple concurrent or sequential treatments may have additive or synergistic effects. The finding that evidence of metastatic disease was associated with a higher number of MSAS symptoms is consistent with a previous report [38]. Future longitudinal studies need to obtain information on specific treatments and metastatic sites and changes in symptom burden over time.

### 4.2 Psychological Cluster

It is not surprising that a psychological cluster was identified because it is the most common symptom cluster across studies of oncology patients [22, 39, 40, 41, 42, 43, 44, 45, 46]. In addition, while the number of psychological symptoms differed [8, 9, 11], this cluster was found in studies of cancer survivors. Consistent with previous studies [9, 11], in the current study, the two symptoms with the highest factor loadings were feeling sad and worrying. Given these consistent findings, as well as the high prevalence rates for depression [47] and anxiety [48] as individual symptoms, as well as co‐occurrence rates for both symptoms of between 12.4% [49] and 45% [50], primary care and oncology clinicians need to perform routine assessments of both symptoms and initiate interventions and/or referrals to psychological support services.

### 4.3 Cancer and Treatment‐Related Cluster

For the cancer and treatment‐related cluster, “I don't look like myself” and hair loss were the two symptoms with the highest factor loadings. While only 17.8% of the sample reported the occurrence of “I don't look like myself”, it ranked as the fourth most severe and distressing symptom. Hair loss was reported by 12.0% of the sample and was ranked as the most distressing symptom. These findings suggest that some survivors experience significant changes in their body image that may warrant counseling.

Weight gain, that was reported by 18.3% of the sample, was the second most distressing symptom. Potential etiologies for weight gain include receipt of hormonal treatments, use of steroids, fatigue and decreases in activity, and/or psychological factors (e.g., stress, anxiety, depression) [51, 52]. Weight gain can have a negative impact on cancer survivors including an increased risk of developing chronic conditions [53], increased risk of cancer recurrence, and increased mortality [51]. Clinicians need to counsel patients to adopt a healthy diet, engage in regular exercise, and seek support to decrease stress and enhance emotional well‐being [54, 55, 56].

Problems with sexual interest or activity, that was reported by 31.6% of the sample, were the most severe and third most distressing symptom. The most common sexual problems in female cancer survivors include vaginal dryness, vaginal stenosis, dyspareunia, decreased libido, and difficulty reaching orgasm [57, 58]. For men, erectile dysfunction, decreased libido, and changes in orgasm and ejaculation are the most common symptoms [57, 58]. These symptoms can occur from cancer treatments themselves; as a result of the side effects of treatment, and/or be associated with psychological distress [59]. As noted in one review [60], common barriers to seeking assistance with sexual problems included patients' embarrassment/discomfort in addressing sexual concerns; the perceived discomfort on the part of clinicians to discuss sexual issues; and limitations within the health care system to address sexual problems. Given the importance of sexual health to survivors' physical and emotional well‐being, clinicians need to initiate and conduct open discussions about their sexual concerns and provide interventions for symptoms and/or referrals.

The remaining symptoms in this cluster (i.e., changes in nails, sweats, feeling bloated, nausea) may be related to the direct effects of current cancer treatments. Over 40% of the current sample was receiving active treatment. Future analyses will compare the number and types of symptom clusters in survivors who were and were not receiving treatment.

### 4.4 Respiratory Cluster

While none of the studies of cancer survivors included respiratory symptoms [7, 8, 9, 10, 11], a respiratory cluster that included shortness of breath and cough was found in our studies of patients with lung [46] and gynecologic [39, 61] cancer, as well as in patients with multiple types of cancer [22, 62]. While a respiratory cluster is expected in patients with lung cancer, prevalence rates for shortness of breath range from 10% to 90% across all types of cancer [63] and was 10.8% in the current sample. Patients can experience this symptom as a result of the cancer itself or from metastatic disease, from treatments that result in pulmonary toxicities, and/or from other cardiopulmonary conditions [64]. Most of the research that evaluated the prevalence of cough assessed patients with lung cancer with reported rates of between 57% [65] and 93% [66]. In a study of the general population of the United States, the weighted prevalence rate for chronic cough was 5.0% [67] which is lower than the 13.4% reported by survivors in the current study. Given that both respiratory symptoms can have a significant impact on survivors' physical and psychological well‐being [65, 67], clinicians need to assess for causes and initiate interventions.

### 4.5 Pain Cluster

While pain was included on the original MSAS, this version added “joint pain” and “muscle aches and pain” to the instrument. In this sample of cancer survivors, pain was the most prevalent symptom (63.7%) and was the ninth most severe and distressing symptom. Equally important, joint pain and muscle aches and pain were reported by 47.7% and 46.3% of the survivors, respectively. The overall prevalence of pain in this study is higher than the 39.8% reported in a systematic review and meta‐analysis of global pain prevalence rates [23]. Given that cancer survivors can experience both cancer and non‐cancer related pain, additional research is warranted on the specific causes of pain and associated interventions.

Joint pain is a common symptom associated with various chemotherapy drugs (e.g., paclitaxel) and hormonal therapies (particularly aromatase inhibitors), as well as other chronic conditions (e.g., osteoarthritis). In fact, consistent with our findings, in a meta‐analysis of aromatase inhibitor–induced arthralgias, the prevalence rate of joint pain in women with breast cancer was 50% [68]. Treatments for this deleterious symptom include acupuncture, exercise, duloxetine, and physical therapy [69].

Muscle aches and pain can result from the administration of chemotherapy (e.g., paclitaxel), growth factors, hormonal therapies, and/or immunotherapies, as well as from electrolyte imbalances and decreased activity [70, 71, 72]. Treatments for this symptom include gentle exercise, non‐opioid analgesics, and complementary therapies (e.g., massage, application of heat or cold).

It is interesting to note that while 37.5% of the sample reported the occurrence of “numbness and tingling in hand/feet”, a symptom associated with chemotherapy‐induced peripheral neuropathy, this symptom did not load on the pain cluster. Given the relatively high prevalence rates for the pain symptoms, future studies need to determine the exact causes of cancer and non‐cancer pain; determine their underlying mechanisms; and identify the most efficacious pharmacologic and non‐pharmacologic interventions.

### 4.6 Weight Loss Cluster

While weight loss and lack of appetite were reported by only about 10% of the current sample, both symptoms can have a negative impact on survivors' health status and treatment outcomes. Unintentional weight loss can occur from nausea, vomiting and taste changes. Alternatively, weight loss can be a sign of the syndrome of cancer cachexia [73]. In the current sample, only 1.8% of the survivors had a BMI that would be classified as underweight (i.e., < 18.5). Clinicians need to monitor changes in survivors' weight; educate survivors about the importance of optimal protein and caloric intake; and refer them for dietary counseling when necessary.

### 4.7 Epithelial Cluster

In the original MSAS, the only epithelial‐related symptoms were changes in skin, itching, and hair loss. However, various cancer treatments (e.g., radiation therapy, immune checkpoint inhibitors) can result in cutaneous effects and associated symptoms [74]. Therefore, in this revision of the MSAS, “changes in skin” was deleted and five cutaneous symptoms were added (i.e., dry skin; calluses on hands and/or feet; changes in nails (brittle); skin rash; swollen, red, and painful sores around the fingernails and/or toenails). While hair loss loaded on the cancer and treatment‐related cluster, skin rash, itching, dry skin, and calluses on the hands and/or feet constituted the epithelial cluster. Given that these cutaneous symptoms were not included in previous studies, this cluster warrants confirmation.

### 4.8 Limitations

Several limitations warrant consideration. Given that the sample was primarily female, white, well‐educated, and had a relatively high annual income, our findings may not generalize to all cancer survivors. In addition, a large proportion of survivors had a diagnosis of breast cancer. Future studies need to evaluate for differences in the consistency and stability of symptom clusters in survivors with different types of cancer. While time since cancer diagnosis was not associated with a higher symptom burden in the multivariable regression analysis, the range in years from diagnosis was large. Future studies need to evaluate for differences in the consistency and stability of symptom clusters in short‐ and long‐term cancer survivors (e.g., less than one year versus greater than one year). Because information is not available on the characteristics of the patients who did not complete the online survey, these findings may underestimate or overestimate cancer survivors' symptom burden. However, this large sample of cancer survivors and the evaluation of 44 symptoms provides benchmark data on their symptom burden.

### 4.9 Conclusions and Implications for Research and Practice

Our findings suggest that six symptom clusters occur in cancer survivors when a comprehensive symptom inventory is administered and EFA is used to extract the symptom clusters. Equally important, survivors experience an average of 10 concurrent symptoms. Survivors who are younger, female, have a higher comorbidity burden, evidence of metastatic disease, and poorer functional status are at increased risk for a higher symptom burden. Additional research is warranted to confirm the prevalence rates for the various symptoms and symptom clusters; identify additional risk factors for a higher symptom burden; and determine the underlying mechanisms for the symptom clusters. Future studies need to develop and test targeted interventions for each of the symptom clusters within and across various types of cancer.

## Author Contributions

**Marilyn J. Hammer:** project administration, investigation, writing, review, visualization. **Carolyn Harris:** writing, review, visualization. **Rachel Pozzar:** project administration, investigation, writing, review, visualization. **Karin Snowberg:** project administration, investigation, writing, review, visualization. **Steven M. Paul:** conceptualization, methodology, supervision, formal analysis, writing, review, visualization. **Bruce A. Cooper:** conceptualization, methodology, supervision, formal analysis, writing, review, visualization. **Maura E. Abbott:** project administration, investigation, writing, review, visualization. **Susan Chang:** writing, review, visualization. **Stacey Kenfield:** writing, review, visualization. **Erin Van Blarigan:** writing, review, visualization. **Katherine Van Loon:** writing, review, visualization. **Jon D. Levine:** conceptualization, methodology, supervision, writing, review, visualization. **Christine Miaskowski:** conceptualization, methodology, supervision, project administration, investigation, formal analysis, writing, review, visualization.

## Funding

The authors have nothing to report.

## Ethics Statement

This study was approved by the University of California San Francisco Institutional Review Board and from the other sites.

## Consent

This study was approved as exempt from requiring written informed consent.

## Conflicts of Interest

The authors declare no conflicts of interest.

## Data Availability

Data are available from Dr. Miaskowski after the completion of a data sharing agreement with the University of California, San Francisco.
